# Cartilage Repair: Promise of Adhesive Orthopedic Hydrogels

**DOI:** 10.3390/ijms25189984

**Published:** 2024-09-16

**Authors:** Peyman Karami, Alexis Laurent, Virginie Philippe, Lee Ann Applegate, Dominique P. Pioletti, Robin Martin

**Affiliations:** 1 Department of Orthopedic Surgery and Traumatology, University Hospital of Lausanne, CH-1011 Lausanne, Switzerland; peyman.karami@epfl.ch (P.K.); virginie.philippe@chuv.ch (V.P.); 2 Laboratory of Biomechanical Orthopaedics, Institute of Bioengineering, School of Engineering, EPFL, CH-1015 Lausanne, Switzerland; dominique.pioletti@epfl.ch; 3 Manufacturing Department, LAM Biotechnologies SA, CH-1066 Epalinges, Switzerland; alexis.laurent@unil.ch; 4 Regenerative Therapy Unit, Reconstructive and Hand Surgery Service, Lausanne University Hospital, University of Lausanne, CH-1066 Epalinges, Switzerland; lee.laurent-applegate@chuv.ch; 5 Center for Applied Biotechnology and Molecular Medicine, University of Zurich, CH-8057 Zurich, Switzerland; 6 Oxford OSCAR Suzhou Center, Oxford University, Suzhou 215123, China

**Keywords:** hydrogel, cartilage, adhesion, ACI, carrier, delivery, therapeutics, integration

## Abstract

Cartilage repair remains a major challenge in human orthopedic medicine, necessitating the application of innovative strategies to overcome existing technical and clinical limitations. Adhesive hydrogels have emerged as promising candidates for cartilage repair promotion and tissue engineering, offering key advantages such as enhanced tissue integration and therapeutic potential. This comprehensive review navigates the landscape of adhesive hydrogels in cartilage repair, discussing identified challenges, shortcomings of current treatment options, and unique advantages of adhesive hydrogel products and scaffolds. While emphasizing the critical need for in situ lateral integration with surrounding tissues, we dissect current limitations and outline future perspectives for hydrogel scaffolds in cartilage repair. Moreover, we examine the clinical translation pathway and regulatory considerations specific to adhesive hydrogels. Overall, this review synthesizes the existing insights and knowledge gaps and highlights directions for future research regarding adhesive hydrogel-based devices in advancing cartilage tissue engineering.

## 1. Introduction

Focal cartilage defects pose significant clinical challenges due to the limited regenerative capacity of cartilage [1]. These defects are often traumatic in origin and typically affect a younger population. The International Cartilage Repair Society (ICRS) classifies them into four grades based on their depth. Most lesions are superficial (grades I-II), producing few or no symptoms and generally responding well to conservative treatment. However, more severe chondral lesions (grade III) involve over 50% of the cartilage thickness, and grade IV lesions can result in a complete loss of cartilage. These advanced defects are associated with significant pain and functional limitations, often necessitating surgical intervention. Chondral damage may also be accompanied by damage to the underlying bone, forming osteochondral lesions. Bone involvement usually occurs secondary to cartilage loss, leading to conditions such as sclerosis and subchondral cyst formation. In some cases, the bone is primarily affected, as seen in osteochondritis dissecans, which can result in the loss of overlying cartilage. While traditional treatments such as microfracture (MF) and mosaicplasty are still widely used for cartilage repair, they are limited to small lesions (<2 cm²) and often yield inconsistent long-term results. These limitations have driven the development of cell-based therapies, such as autologous chondrocyte implantation (ACI). ACI offers several advantages, including the production of hyaline-like cartilage, long-term efficacy, and the ability to treat larger lesions [2,3].

**Figure 1:**
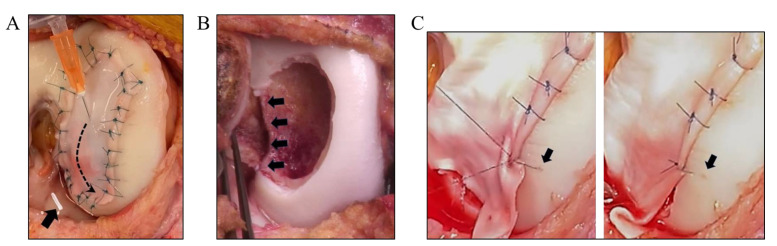
Some existing critical challenges in therapeutic delivery and retention in cell-based therapies, such as (**A**) leakage of cell suspension between sutures and weak performance of fibrin glue, (**B**) application in uncontained defects, and (**C**) suturing complexity to surrounding cartilage: tissue tearing from suture tightening.

In cell-based therapies for cartilage repair, the ideal carrier for delivering therapeutic agents (e.g., autologous chondrocytes, stem cells, growth factors) remains a topic of debate among researchers and clinicians [4]. In the first-generation ACI, a periosteal flap was used to cover the defect and implanted cells. This technique has evolved to second-generation autologous grafting due to the risk of graft hypertrophy and the relative invasiveness of the procedure. In second-generation ACI, collagen membranes are sutured over the defect site to secure the cultured cells in place. We have recently introduced the second-generation ACI at the Lausanne University Hospital with a specialized culture medium using human platelet lysate (hPL) [5,6]. This medium was designed to optimize human articular chondrocyte proliferation and functionality for clinical implantation. We showed that the cells cultured with hPL in monolayer exhibit similar growth characteristics to those cultured with autologous human serum (aHS), including cell morphology and growth rates. We selected the Chondro-Gide membranes due to their widespread use in the field to date [7]. Although this generation addressed some negative issues of the first-generation ACI, especially in larger defects, challenges remained with cell retention and distribution [2]. Once the cell suspension was injected under the membrane, fibrin glue was often used to seal the injection site and prevent leakage of cells [8]. However, ensuring a proper seal, especially without excessive application of glue, is a surgical challenge, especially for poorly contained defects. Moreover, providing the balance between tightness and gentleness of the membrane is hard to achieve in order to maintain the integrity of both the membrane and the underlying cells. Uneven tension can cause gaps or folds in the membrane, and despite careful suturing, there is always a risk of chondrocyte suspension leaking, which can significantly reduce the number of cells available for repair and affect the clinical outcome. Many chondral defects have limited visibility and access, which presents additional difficulties in cell delivery, and sutures applied on the edges might shear (See Figure 1). 

In third-generation ACI, carriers such as 3D scaffolds, membranes, beads, and injectable matrices offer a structured environment that can potentially enhance tissue formation [9]. However, these approaches are associated with unresolved challenges, such as initial consistency, application in uncontained defects, stability of the graft during the initial healing phase, and uniform cell distribution. The positioning of the cell-loaded scaffold is key to achieving optimal coverage and integration within the cartilage defect site. As there are irregular variations in size and depth of cartilage defects, the scaffold is often customized or trimmed during implantation to fit specific contours of the defect site [10]. Despite using fibrin glue to enhance stability, securing the implanted scaffold with structural integrity is challenging, particularly in high-load bearing areas. Although use of small beads and microcarriers can result in delivering a high density of cells or bioactive agents with a controlled release of therapeutics over time [11,12], they present the risk of limited stability, uneven distribution, and potential migration from the target site.

In particular, integration of the implanted components with the surrounding native cartilage and underlying bone is pivotal in the repair process [13]. Clinically, this is critical for ensuring the durability and functionality of repair. Lateral integration refers to the ability of the new cartilage to bond seamlessly with the adjacent native cartilage. Weak lateral integration can lead to weak interfaces and failure points. Although not commonly highlighted in clinical discussions, this biological concept is essential for the long-term stability and functionality of repaired cartilage. Integration with the subchondral bone is also crucial to provide stability to the repaired area. Innovative scaffold designs and biomaterials are therefore required to address these challenges effectively.

**Figure 2:**
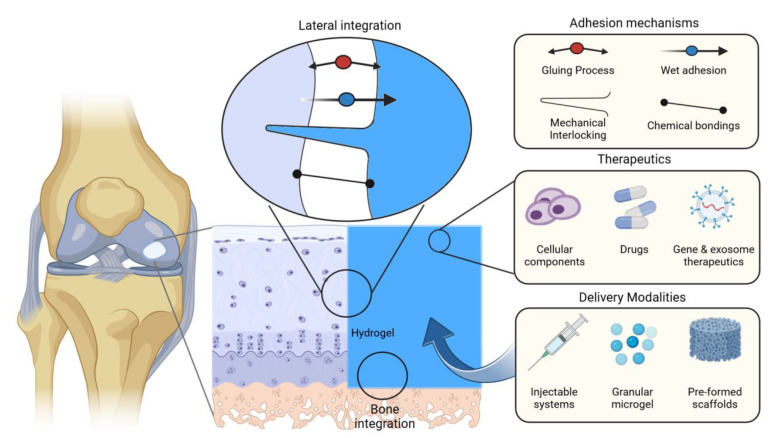
Schematic illustration of adhesive hydrogels as promising therapeutic carriers in cartilage repair and related tissue integration mechanisms. Various mechanisms for promoting the adhesive performance of hydrogel systems, therapeutic options, and hydrogel delivery methods are shown.

Despite decades of research and a pressing clinical demand for enhanced treatments for cartilage lesions, the approval of delivery products for cartilage repair and regeneration has been scarce [14]. Recently, adhesive hydrogels have shown great potential in cartilage tissue engineering. The ability of these materials to adhere to the defect site without the need for extensive suturing could potentially facilitate integration with surrounding tissue and reduce the risk of displacement (Figure 2). This in turn reduces the risk of cell leakage and promotes uniform tissue formation. By mimicking the native extracellular matrix (ECM) environment, hydrogel scaffolds could provide a conducive microenvironment for cell growth and tissue regeneration. These water-swollen materials could enhance cell containment and distribution, provide mechanical support during the healing process, and offer controlled degradation rates [15,16]. These innovative materials could address some of the existing challenges in the cartilage therapies mentioned above.

In this review, we present the potential of adhesive hydrogels in addressing the challenges of repairing cartilage lesions. We will discuss current treatment limitations, principles of cartilage tissue engineering, and how adhesive hydrogels could offer unique benefits for cartilage repair. By highlighting recent advancements, we aim to provide insights into future directions for research and the clinical advancement of adhesive orthopedic hydrogels.

## 2. Existing Treatments and Challenges in Cartilage Repair

Current treatments for cartilage repair encompass a range of approaches, including bone marrow stimulation (BMS) techniques, osteochondral autograft transfers, osteochondral allograft transplantation, and different generations of ACI. While these treatments have shown varying degrees of success in alleviating symptoms and restoring function, they are not without limitations [17].

Today, BMS remains the most frequently used surgical method in cartilage repair, mainly due to lower costs and surgical simplicity compared to exogenous cell therapies. As a less invasive procedure, it involves creating small holes in the bone beneath the damaged cartilage to stimulate the formation of reparative tissue [3]. BMS techniques have evolved from the microfracture method developed by Pridie in the 1950s and later advanced into microperforation by Steadman in the 1980s [18]. Augmented microfracture is a more recent development that incorporates a biocompatible scaffold or matrix over the defect area to improve the stability of the induced blood clot and tissue quality. In 2003, AMIC was introduced by Behrens, in which a collagen I/III membrane was used to capture cells released from the drill holes, leading to the formation of a more pronounced fibrocartilaginous layer [19].

Furthermore, three generations of ACI have been developed, with the second- and third-generation being most commonly used. First-generation ACI was initially introduced by Brittberg et al. in 1987. As a two-step procedure, it involves isolating cells from a small piece of healthy cartilage, amplifying them in vitro, and injecting a suspension of cultured autologous chondrocytes under a periosteal flap harvested from the tibia [20,21]. Second-generation ACI (C-ACI) utilized a collagen membrane to improve cell containment and reduce hypertrophy [22]. In particular, ACI therapies are more effective in areas subjected to lower mechanical constraints, unlike the high-stress regions such as the patellofemoral joint. Matrix-induced ACI (MACI), the third-generation technique, was introduced in the early 2000s and involved seeding cells onto a 3D scaffold with specific incubation periods to support early cell differentiation before implantation and address the challenges associated with arthroscopic implantation and the need for sutures. Although there is a tendency towards using third-generation ACI, an efficient delivery system remains a surgical issue, as discussed earlier.

Moreover, in the available therapeutic approaches, parameters such as incomplete tissue remodeling, scaffold degradation, and inadequate cellular infiltration hinder the formation of strong tissue interfaces between the repair site and surrounding healthy tissues. Such factors may result in uneven integration patterns, leading to potential weak points and areas of vulnerability within the repaired cartilage. These limitations are particularly significant in classical bone marrow stimulation techniques and autologous osteochondral grafting, where deterioration in outcomes has been observed after two years, especially in younger patients [2]. The use of scaffolds or matrices introduces additional complexities, as scaffold degradation and potential mismatches in scaffold properties can disrupt tissue integration and compromise mechanical stability at the interface. Furthermore, the formation of fibrous or fibrocartilaginous tissue instead of hyaline-like cartilage further hinders successful outcomes. In Table 1, a comparison of existing treatments for cartilage repair is provided.

## 3. State of the Art in Adhesive Hydrogels

Hydrogels are 3D crosslinked networks of polymers with high water content that can be designed to provide a wide range of physicochemical and biological properties. These materials have the ability to mimic the structural and biochemical properties of native ECM [32].

### 3.1. Hydrogel Composition

There are three categories of hydrogels for cartilage tissue engineering applications based on polymer type, including natural, synthetic, and hybrid hydrogels (See Table 2). Natural hydrogels (e.g., gelatin, hyaluronic acid, chitosan, etc.) are derived from biological sources and offer intrinsic bioactivity and biocompatibility. This makes them attractive choices for supporting cell adhesion, proliferation, and differentiation [33]. However, their poor mechanical properties and fast degradation kinetics may require optimization to match those of native cartilage tissue. Various strategies such as chemical cross-linking, blending with other reinforcing materials (e.g., collagen or silk fibroin), and incorporating bioactive molecules could be employed to enhance the performance of natural hydrogels. Such strategies could facilitate maintaining the biocompatibility of the scaffold while enhancing its mechanical and biological properties. Some natural hydrogels can be supplemented with bioactive compounds derived from natural sources, such as natural antioxidants. These compounds help reduce inflammation in the injured area and promote tissue regeneration.

Synthetic hydrogel components (e.g., polyethylene glycol (PEG), poly(N-isopropylacrylamide) (PNIPAAm), and poly(vinyl alcohol) (PVA), etc.) offer precise control over mechanical and biochemical properties [34]. These materials can be functionalized with cell-adhesive peptides, growth factors, or other bioactive molecules to enhance cellular interactions and tissue regeneration. In hybrid hydrogels, natural and synthetic components are combined to synergize the advantages of both materials and to minimize their limitations.

Marine-derived hydrogels, such as those based on alginate, agarose, and chitosan, provide distinct biochemical and mechanical properties that can be advantageous for cartilage repair. For instance, alginate and agarose are known for their biocompatibility and ability to form hydrogels that mimic the extracellular matrix, supporting cell growth and tissue regeneration effectively [35]. Mussel-derived bioadhesives have gained attention in biomedical research due to their strong adhesion properties, even in moist environments. These bioadhesives, inspired by the adhesive proteins used by mussels to attach to wet surfaces, can be incorporated into hydrogel formulations to enhance their stability and attributes of adhesion to tissue surfaces [36].

While adhesive hydrogels offer unique advantages in terms of tissue adhesion and integration, their degradation kinetics, like those of other hydrogels, should be tailored to meet specific therapeutic requirements, ensuring that they provide the necessary support during the healing process.

#### Clinical Impact

Clinically, although natural hydrogels promote physiological healing processes [37], the consistency and reproducibility of synthetic hydrogels provide predictable performance in surgical outcomes. However, the lack of inherent bioactivity and potential for foreign body reactions necessitates thorough evaluation to avoid adverse reactions.

Specifically, hydrogels should exhibit controlled degradation kinetics to match tissue regeneration and remodeling rates. Rapid degradation may compromise structural integrity, while slow degradation may limit cell infiltration and tissue integration [38]. Natural hydrogels typically degrade through enzymatic processes. For example, collagen is broken down by collagenases, while HA is degraded by hyaluronidases. Although the degradation attributes can be tuned through crosslinking or blending, the exact in vivo degradation rates remain less predictable. Synthetic hydrogels degrade through hydrolytic or enzymatic pathways, depending on their chemical structure. Although these materials offer controlled degradation profiles tailored by their composition, the long-term biocompatibility and the potential accumulation of degradation by-products require further investigation.

**Table 2:** Comparison of natural, synthetic, and hybrid hydrogels for cartilage tissue engineering.

Hydrogel Type	Examples (Source)	Pros	Cons	References
Natural Hydrogels	Alginate (Marine, Algae) Collagen (Animal) Hyaluronic Acid (Animal or Bacterial) Chitosan (Marine, Crustacean) Gelatin (Animal) Fibrin (Animal) Cellulose (Plant)	- Bioactivity and biocompatibility - Biodegradation - Supports cell adhesion, proliferation, and differentiation - Anti-inflammation and antioxidant	- Poor mechanical properties - Unpredictable degradation kinetics - Potential for immunogenicity	[33,39]
Synthetic Hydrogels	Polyethylene Glycol (PEG) Poly(N-isopropylacrylamide) (PNIPAAm) Poly(vinyl alcohol) (PVA) Poly(lactic-co-glycolic acid) (PLGA) Polycaprolactone (PCL)	- Precise control over mechanical and biochemical properties - Customizable scaffold design - Reproducible	- Risk of foreign body reaction - Poor biological activity - Uncertain long-term biocompatibility	[34,40]
Hybrid Hydrogels	Combinations of natural and synthetic components	- Synergizes advantages of both natural and synthetic materials - Balances bioactivity and mechanical strength	- Complexity in design and synthesis - Potential for uneven degradation or integration	[40,41,42]

### 3.2. Adhesion Mechanisms of Hydrogels

Importantly, hydrogel-tissue adhesion represents a critical aspect of biomedical applications and affects the success of various therapeutic interventions. The adhesion process is the initial attachment of the hydrogel to tissue (chemical concept), while integration refers to the long-term incorporation of the hydrogel into the tissue. Clinically, adhesion is essential for immediate stability, particularly in the challenging environments of bone and cartilage interfaces. Therefore, to reach long-term integration, sufficient initial adhesion is essential. Indeed, tissue integration is a gradual process influenced by parameters such as initial adhesion strength, biological environment, healing capacity, and mechanical stresses.

The adhesion of hydrogels is influenced by hydrogel-tissue interfacial energy and the capability of hydrogel networks for energy dissipation during deformation [43,44]. Interfacial energy is determined by the surface characteristics and the interaction forces, while mechanical dissipation reduces stress concentration, prevents crack propagation, and maintains adhesion under dynamic conditions. The synergy between high interfacial energy and efficient mechanical dissipation enables hydrogels to adhere strongly to the tissue surface [44,45].

Adhesion processes are influenced by surface chemistry, topology, and the physicochemical properties of the hydrogel [46]. There is no direct relation between the type of polymer (composition, see Section 4.1) and adhesion. Bone adhesion requires the material capability to anchor to a hard and mineralized surface, while cartilage adhesion relies on interactions with ECM components, promoting a smooth integration with surrounding tissue. Lateral integration with cartilage ensures a seamless joint surface, while bone integration provides necessary mechanical support. Effective hydrogel designs must therefore optimize both adhesion and integration to enhance clinical outcomes in cartilage repair.

**Figure 3:**
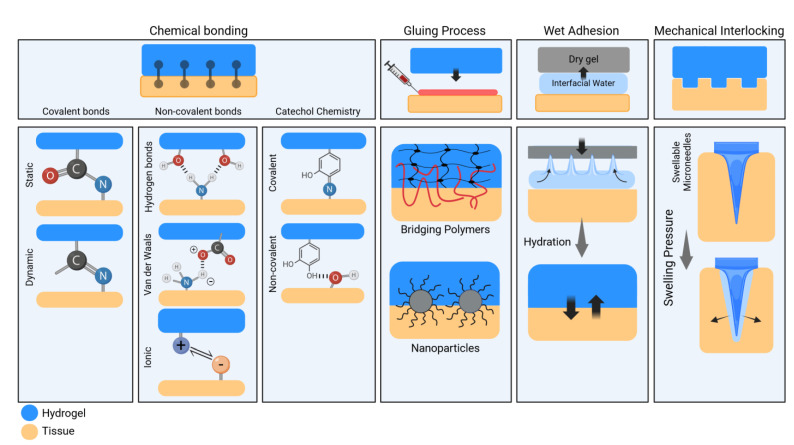
Classification and mechanisms of hydrogel adhesion to tissues with representative examples. Explanations of the adhesive aspects are reported in the text.

Adhesive hydrogels can be systematically classified into four categories based on their interfacial adhesion principles (see Figure 3).

#### 3.2.1. Chemical Bonding

Hydrogels in this category achieve adhesion through chemical interactions between functional groups present on the hydrogel surface and complementary moieties within the cartilage tissue substrate [47,48,49,50]. Usually, one or more mechanisms can be employed to create chemical bonding at the interfaces. The total bonding energy should be sufficient to maintain the hydrogel securely in place, regardless of the specific bonding type or mechanism used.

##### Covalent Bonding

These bonds are crucial in adhesion processes with both static (irreversible) and dynamic (reversible) bonds. An example of this is the creation of covalent amide bonds using carbodiimide/hydroxysuccinimide coupling chemistry, which leads to strong and irreversible adhesion between carboxylates on hydrogel chains and primary amine groups on tissue surfaces. Additionally, dynamic covalent bonds, such as Schiff’s base linkages, enhance adhesion strength, allowing hydrogels to stick to various wet tissue surfaces [51].

##### Non-Covalent Interactions

Non-covalent or supramolecular interactions, which do not involve the sharing of electrons, also contribute to hydrogel-tissue adhesion. These interactions include hydrogen bonding, van der Waals forces, electrostatic, hydrophobic, and host-guest interactions. While considered weaker than covalent bonds, supramolecular interactions can still result in strong adhesion. For example, hydrogen bonds and van der Waals forces are important in adhesion mechanisms based on functional groups of hydrogel backbone polymers and tissue surfaces. Electrostatic interactions, on the other hand, involve the attraction or repulsion of charged moieties, which can be harnessed in electro-adhesion processes for tissue adhesion [52,53].

##### Catechol Chemistry

Inspired by marine animals and that seen with mussel adhesion, catechol chemistry has emerged as a promising approach for fabricating tissue-adhesive hydrogels [54,55]. The remarkable adhesion ability of mussels onto various substrates is mediated by the secretion of an amino acid called 3,4-dihydroxyphenylalanine (DOPA), which contains catechol groups [56]. Synthetic molecules resembling the catechol structure of DOPA, such as dopamine (DA) and tannic acid (TA), have been successfully incorporated into hydrogel formulations to mimic mussel adhesion mechanisms. These adhesion mechanisms involve a combination of covalent (e.g., Schiff base, Michael addition) and non-covalent bonds (e.g., hydrogen bonds, π-π stacking, metal coordination) between catechol and functional groups on tissue surfaces.

#### 3.2.2. Interfacial Gluing

Interfacial gluing remains a common technique for achieving adhesion between hydrogels and tissue surfaces. By directly applying adhesive substances at the interface, these solutions flow and diffuse bidirectionally, penetrating microstructures [51]. Once cured, typically after a specific time-period, the adhesive forms a binding bridge between the two surfaces.

Various polymers, known as bridging polymers, have been explored for topological adhesion, including chitosan, alginate, carboxymethyl cellulose, PAA, and polyacrylamide (PAAM) [57]. Researchers have developed rapid, tough double-network hydrogels utilizing chitosan as the bridging polymer, resulting in strong tissue adhesion attributed to interpenetrated chitosan macromolecules that easily diffuse and entangle with hydrogel chains and tissue fibers.

Another similar approach can be implemented by using adhesive nanoparticles. Silica nanoparticle solutions have been introduced as a glue between hydrogel and tissue surfaces. Adhesion mechanisms rely on the chemical adsorption of hydrogel polymeric chains and tissue fibers onto the nanoparticle surface, forming bridges between nanoparticles. These bridges are held together by reversible chemical van der Waals forces, allowing continuous detachment and re-adsorption of hydrogel chains and tissue fibers under tension, preventing chain breaking and ensuring sufficient hydrogel-tissue adhesion [58,59].

#### 3.2.3. Wet Adhesion

In biomedical adhesion, overcoming the challenges posed by wet surfaces is critical. Wet adhesion, influenced by mechanisms such as surface tension, capillary force, and Van der Waals interactions, emerges as an effective approach to address this challenge. Recent approaches involve the development of dry double-sided tape (DST) formulations [60]. These tapes swiftly adhere by removing interfacial water and initiating temporary crosslinking, followed by covalent bonding with tissue amine groups. Similarly, hydrogel tapes utilize catechol chemistry, gradually forming covalent bonds with tissue amino groups over time. This dual-stage adhesion mechanism offers instant bonding and long-term stability, making these tapes promising for diverse biomedical applications. Furthermore, engineered surface geometries or patterns can enhance adhesion to tissue substrates by topo-geometrical patterning. These patterns may include microscale or nanoscale features designed to maximize contact area, promote tissue ingrowth, or optimize mechanical coupling between the hydrogel and the tissue interface. Again, taking other efficient biological mimicking examples with gecko-inspired micropillars, adhesion maintenance in wet conditions can be fabricated from hydrophilic materials such as PEGDMA. Similarly, tree frog hexagonal foot pad patterns, with microgrooves for water expulsion, and clingfish’s hexagonal geometrical features are translated into tissue-adhesive hydrogels [61]. These bioinspired designs enhance underwater adhesion, demonstrating effective interface interaction between hydrogel and substrate, and thus are perfect biological examples for bioengineering mimicking.

#### 3.2.4. Mechanical Interlocking

Adhesive hydrogels employing mechanical interlocking mechanisms rely on physical entanglement or interlocking structures to anchor the hydrogel to the tissue substrate. The hydrogel interface’s microscopic features or surface roughness enhance mechanical interlocking, facilitating adhesion to tissue surfaces [62,63]. For instance, taking inspiration from parasites such as *Pomphorhynchus laevis*, researchers developed microneedles with swellable hydrogel tips. These tips, coated with a poly(styrene)-block-PAA copolymer hydrogel, absorb tissue fluids, swell, and enhance adhesion significantly compared to non-coated microneedles [62].

The formation time of hydrogels is affected by various factors such as hydrogel network design, curing process, reaction conditions, applied energy, and chemical modifications. For example, in light-curable hydrogels, the curing process kinetics rely on the defined photoinitiator type and concentration and light source specifications. The intensity and duration of light exposure directly affect the polymerization and the obtained system properties [64].

#### 3.2.5. Clinical Impact

Understanding the adhesion mechanisms of hydrogels is required for successful application in clinical settings. Effective adhesion impacts both the immediate stability of implants and long-term outcomes. If a hydrogel is not correctly positioned or fully cured at the cartilage defect site, it may fail to adhere properly or provide poor coverage, which can impair repair and integration. Wrong positioning may also lead to displacement or degradation, and ultimately mechanical damage or loss of functionality. In injectable hydrogels, it is essential to control the precursor flow and appropriate placement. The flow of sterile saline during surgical procedures is challenging, as it can displace or dilute the hydrogel; however, this can be managed by using viscosity-enhancing additives or employing techniques to temporarily dry the defect site before application. Also, optimizing the curing process can ensure that the hydrogel sets correctly.

In recent years, advancements in hydrogel fabrication techniques have enabled the development of various customizable and functional scaffolds with spatial control over mechanical and biochemical cues. Despite significant advancements, challenges remain in optimizing the adhesive properties of hydrogels to allow for a perfect interface creation and to achieve seamless integration with native cartilage tissue. Factors such as scaffold geometry, surface chemistry, and mechanical properties influence the adhesion kinetics and stability of hydrogel-tissue interfaces. Moreover, the dynamic nature of cartilage tissue presents additional complexities.

### 3.3. Adhesive Hydrogels for Constituent Delivery

Adhesive hydrogels offer a solution for acting as carriers to deliver therapeutic agents and cellular components and facilitate tissue regeneration. Current research in this field encompasses diverse strategies categorized based on the constituents incorporated within hydrogel matrices [65]. Herein, we delineate these investigations, classifying them based on the constituent type encapsulated within the hydrogels, and discuss their implications for cartilage repair management (Table 3).

#### 3.3.1. Hydrogels with Therapeutic Agent Incorporation

These hydrogel-based drug delivery systems offer sustained release of therapeutic agents, targeting key pathways involved in cartilage repair and inflammation modulation [66,67]. Hydrogels laden with therapeutic agents, including non-steroidal anti-inflammatory drugs (NSAIDs), corticosteroids, and inflammation-modulating drugs, have garnered significant attention for their potential to alleviate pain and inflammation. While NSAIDs and corticosteroids offer symptomatic relief, concerns regarding adverse effects necessitate alternative delivery strategies. Emerging research explores the encapsulation of methotrexate, hydroxychloroquine, and other anti-inflammatory agents within hydrogel matrices to mitigate side effects and enhance therapeutic efficacy. These drug-loaded hydrogels hold promise for sustained drug release, localized delivery, and improved patient outcomes in cartilage defect treatment.

#### 3.3.2. Hydrogels with Cellular Components and Various Cell Types

Incorporating cellular sources and their components, such as chondrocytes, mesenchymal stem cells (MSCs), and cytokine cocktails, into hydrogel scaffolds as supportive microenvironments facilitates the formation of functional cartilage tissue [16,68]. Primary articular chondrocytes are the most widely used cells for regenerating hyaline cartilage, but acquiring sufficient autologous cells remains a key limitation [69]. In vitro cell expansion risks the irreversible loss of the chondrogenic phenotype; therefore, growth factor supplementation to maintain chondrocyte tissue formation capacity might be required [26]. Non-articular chondrocytes from sources such as costal and nasal cartilage are investigated as alternatives due to their similar biochemical properties to articular cartilage. Nasal chondrocytes, for instance, show promising results in clinical trials for cartilage repair in Europe [70]. Studies have demonstrated the efficacy of MSCs in reducing inflammation, promoting chondrogenesis, and enhancing tissue repair in preclinical models. Furthermore, the incorporation of specific cytokines and peptides within hydrogel formulations holds the potential for modulating the local microenvironment, stimulating tissue regeneration, and attenuating disease pathology [71]. Platelet-rich plasma (PRP) taken from the standardized blood of the patient has also been combined with hydrogels and used as an enhanced visco-supplementation option to reduce cartilage degradation in the clinical setting [72,73]. These cell-laden and/or biological component hydrogels offer a multifaceted approach to address the complex pathophysiology of the lesion and foster cartilage repair.

#### 3.3.3. Gene Therapy and Exosome Therapeutics

Gene therapy and exosome-based approaches hold promise to enhance the regenerative potential of adhesive hydrogels [74]. Gene delivery systems encapsulating transcription factors such as Sox9 aim to promote chondrocyte differentiation and cartilage formation. Similarly, exosomes derived from MSCs exhibit regenerative properties, modulating immune responses and facilitating tissue repair. Integrating gene vectors or exosome payloads within hydrogel matrices enables targeted delivery and sustained release, augmenting reparative processes, as long as the manufacturing process of the complex biologicals is well standardized.

**Table 3:** Overview of various components used in adhesive hydrogels for cartilage tissue engineering. The table categorizes different components, including drugs, cells, cytokines, peptides, platelet-rich plasma (PRP), genes, and exosomes, commonly incorporated into adhesive hydrogels for cartilage tissue engineering applications.

Component	Classification	Function	Examples/References
Therapeutic agents	NSAIDs	Alleviate pain and inflammation, reduce joint swelling, and inhibit osteoarthritis (OA) progression	Ibuprofen [75], Naproxen [76], Celecoxib [77], Methotrexate [78], and Hydroxychloroquine [79]
	Corticosteroids	Alleviate pain and inflammation, reduce joint swelling, and inhibit OA progression	Prednisone [80], Dexamethasone [81], and Triamcinolone [82]
Cellular sources and Components	Cells	Promote tissue regeneration, reduce inflammation, and enhance tissue repair	Articular chondrocytes, Nasal chondrocytes, Mesenchymal Stem Cells (MSCs), Adipose-derived Stem Cells (ASCs) [83], and Progenitor cells [84]
	Cytokines	Promote cartilage regeneration	Fibroblast growth factor (FGF), TGF-β [85]
	Peptides	Promote cartilage regeneration	CK2.1 [86]
	Platelet-Rich Plasma	Promote cartilage regeneration, reduce inflammation, and enhance tissue repair	Concentrated platelets [87]
Gene therapy and Exosome delivery	Transcription Factors	Enhance chondrocyte differentiation and promote tissue repair	Sox 9 [88]
	Gene Vectors	Enhance chondrocyte differentiation and promote tissue repair	Lentiviral vectors, recombinant adeno-associated virus (rAAV) [89]
	MSC-Derived Exosomes	Modulate immune response and enhance tissue regeneration	MSC-derived exosomes [90]

### 3.4. Hydrogel Delivery Modalities

In addition to the constituents encapsulated within the hydrogels, the delivery modalities are important in determining their efficacy and applicability in clinical settings. Adhesive hydrogel systems can be delivered or implanted using different modalities, including injectable [91], granular [92], and preformed systems [93].

#### 3.4.1. Injectable Hydrogels

Injectable hydrogels are parenteral formulations that can be delivered minimally invasively through needles or catheters into the target site. Injectable hydrogels offer advantages in terms of delivery simplicity, adaptability to irregularly shaped defects, and minimally invasive delivery, making them suitable for arthroscopic procedures and other intra-articular applications [94]. As an injectable system, a poly(ethylene glycol) diacrylate (PEGDA) hydrogel, developed by Sharma et al., combined with chondroitin sulfate adhesive, demonstrated efficacy in supporting cartilage matrix production and enhancing tissue repair in articular defects. Preclinical and pilot clinical studies showed improved tissue filling, reduced pain, and enhanced knee function [95].

#### 3.4.2. Granular Hydrogels

Granular hydrogels consist of hydrogel particles or granules that can be packed into defects or cavities to fill void spaces and promote tissue regeneration [96,97]. These granules can be injected or implanted directly into the defect site, where they conform to the irregular contours of the tissue and promote cell infiltration and matrix deposition. Zhu et al. developed a granular hydrogel composed of hyaluronic acid, polyethylene glycol, and gelatin. This approach, involving photo-annealing post-injection, facilitates chondrocyte expansion and maintains their phenotype, leading to enhanced hyaline-like cartilage regeneration [92].

#### 3.4.3. Preformed Hydrogels

Preformed hydrogels are fabricated ex vivo into specific shapes or sizes before implantation. These hydrogels are typically molded or cast into the desired geometry and then implanted into the defect site. Preformed hydrogels provide precise control over the hydrogel structure and properties and facilitate tailored designs for patient-specific applications. Wei et al. developed an approach utilizing 3D printing technology and preformed adhesive hydrogels to replicate the tri-layer structure of articular cartilage, offering lubrication, load-bearing, and adhesive fixation functions. In vitro data has shown that the adhesive layer at the bottom of the composite scaffold provided attachment to the subchondral bone component [93].

## 4. Lateral Integration, an Unmet Need in Carriers for Cellular Therapy in Cartilage Defects

As discussed earlier, achieving effective integration between repaired cartilage and surrounding tissue, especially lateral integration, remains a significant challenge. Despite advancements, developing an adhesive product that ensures robust lateral integration remains elusive. Adequate integration is crucial for restoring joint surface continuity, maintaining biomechanical properties, and preventing secondary cartilage degeneration. Importantly, insufficient integration can lead to biomechanical mismatches, increased friction, and impaired joint function. Therefore, challenges persist in establishing stable interfaces capable of withstanding physiological loading and maintaining structural integrity. The absence of effective strategies for promoting lateral integration presents a substantial barrier to the clinical translation of cartilage tissue engineering approaches.

Lateral integration is crucial for halting the progression of tissue degeneration. The presence of fissures and vertical cracks as structural imperfections in the affected cartilage [98] could impair cartilage mechanobiology and diminish its ability to bear loads effectively [99].

**Figure 4:**
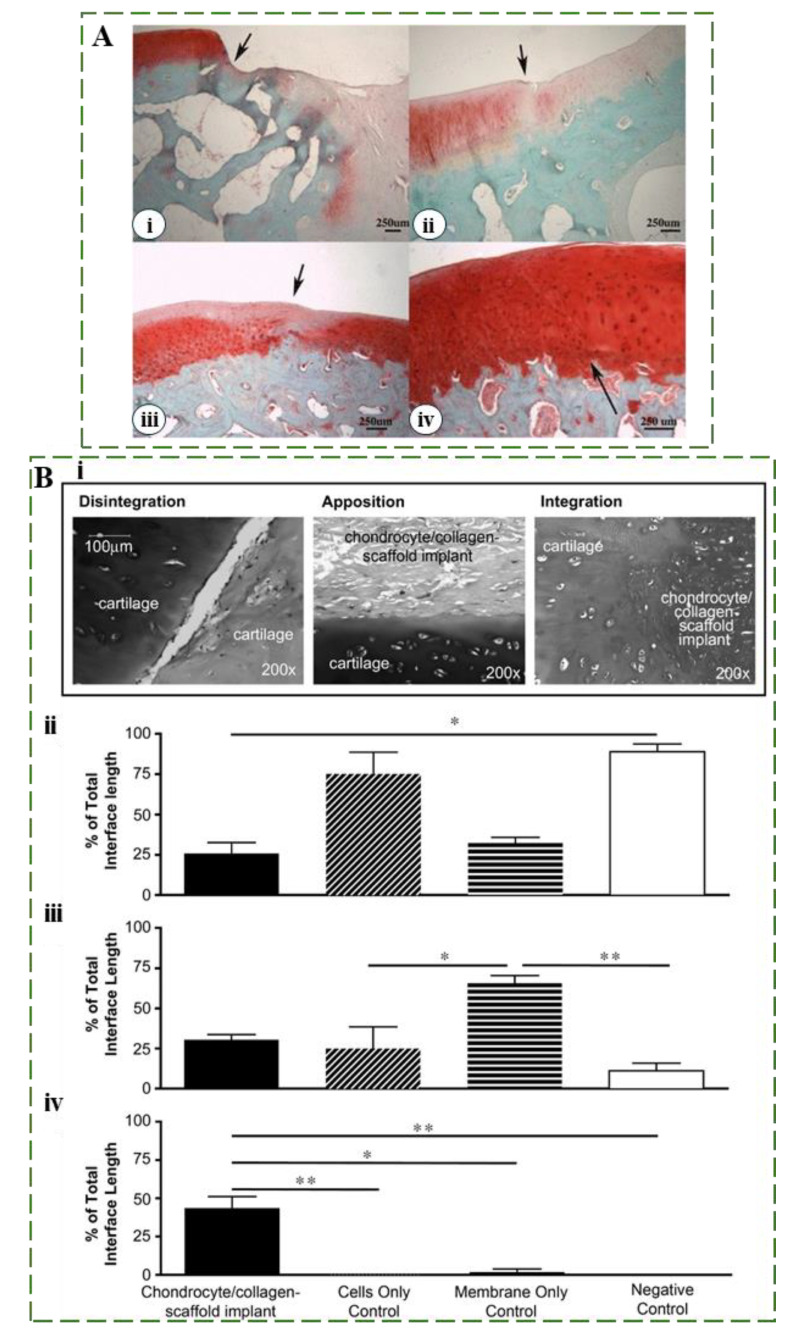
(**A**) Representative Safranin O-stained sections depicting varying grades of integration with native cartilage. Black arrows highlight the junction area: (i) Poor integration, no tissue fusion; (ii) Integration with hypocellularity and surface fissuring; (iii) Enhanced integration with residual hypocellularity; (iv) Excellent integration; Reproduced with permission from Ref. [101], Copyright 2014, Springer. (**B**) Schematic representation of integration examination for repaired and native cartilage. Cartilage integration facilitated by a chondrocyte/collagen-scaffold implant system: Chondrocytes seeded onto a collagen membrane formed the implant, positioned between two cartilage discs. Histomorphometric analysis compared how well different groups integrated along the interface after 40 days: implanted chondrocytes, cells-only, membrane-only, and negative controls. Integration quality was categorized into disintegration, apposition, and integration percentages across the interface length. Statistical analysis was reported using the Kruskal–Wallis non-parametric ANOVA, fol-lowed by the Mann–Whitney U-test with a Dunn post hoc correction for multiple compar-isons. * *p* < 0.05; ** *p* < 0.01. Comparisons not marked with an asterisk are not statistically significant. Reproduced with permission from [102], Copyright 2009 Elsevier Ltd.

Ensuring stable and long-term lateral integration is crucial in cartilage repair strategies, as initial integration does not always ensure lasting stability. The nature of the chondral repair tissue often transitions to a suboptimal fibrocartilaginous state, with altered collagen ratios and reduced proteoglycan contents, diminishing its resemblance to healthy hyaline cartilage. As shown by Shapiro et al., despite initially fusing with host tissue, the inherent biomechanical weaknesses of fibrocartilaginous tissue make it prone to developing microfractures along the lateral margins between the host and repair sites. These microfractures progress into deeper fissures over time, ultimately leading to the deterioration of the repair tissue [100]. As shown in Figure 4A(i-iv), various degrees of integration are observed, ranging from initial fusion with host tissue to high-quality integration [101]. Moreover, quantifying the extent of integration is important for assessing the efficacy of cartilage repair interventions. Figure 4B provides a schematic representation of a chondrocyte/collagen-scaffold implant system, offering visual insights into the experimental framework used to examine the dynamics of integration quality. Researchers can quantitatively evaluate parameters such as disintegration, apposition, and integration at the repair interface (Figure 4B(i-iv)). Thus, an enhanced understanding of the integration process guides the development of strategies to improve integration outcomes [102].

### 4.1. Mechanisms Leading to Problems in Lateral Integration

Lateral integration challenges arise from the complex nature of cartilage tissue, where cellular parameters, tissue origin, and ECM composition influence the ability of the repair tissue to effectively merge with surrounding cartilage [103,104]. Moreover, variations in cellularity, matrix turnover, and metabolic activity within the joint microenvironment further complicate the integration process.

Several factors impede lateral integration during cartilage repair (See Table 4). Cellular factors such as chondrocyte viability and phenotype, influenced by various intrinsic and extrinsic factors, significantly impact tissue integration [105]. Donor-related factors, including age and tissue origin, further complicate the process with age-related declines in chondrocyte function and variations in tissue biosynthetic capacities hindering integration efforts. The ECM, particularly the collagen network and the presence of proteoglycans, creates additional barriers to chondrocyte migration and integration. Moreover, biomaterial properties and scaffold integration processes play crucial roles, influencing repair outcomes and lateral integration success. Understanding these multifaceted mechanisms is essential for developing effective strategies to overcome integration challenges and improve cartilage repair outcomes.

### 4.2. Strategies for Promoting Lateral Integration

In response to the challenges of achieving stable lateral integration, researchers and clinicians have explored various strategies to improve integration between repaired and native cartilage [106]. Enzymatic treatments, such as collagenase digestion, have shown promise in promoting tissue remodeling and enhancing collagen deposition, thereby facilitating better integration [107,108]. Additionally, researchers have explored methods such as treatment with apoptosis inhibitors to prevent chondrocyte death at wound edges [105]. Delivering exogenous chondrocytes to the cartilage interface, either suspended in fibrin glue or seeded onto a collagen membrane, shows potential for enhancing integration [102,109]. Optimizing scaffold properties and incorporating growth factors into the repair process are other strategies employed to enhance lateral integration. Fine-tuning scaffold composition and architecture allows for the creation of an environment conducive to chondrogenic differentiation and matrix production, fostering improved integration between repair tissue and surrounding cartilage. Moreover, the targeted delivery of growth factors and cell sources hold promise for promoting tissue maturation and enhancing integration. Table 5 presents an overview of the strategies for lateral integration enhancement.

Despite these efforts, achieving robust lateral integration in cartilage repair remains a significant clinical challenge. The complex interplay of cellular, matrix-related, and environmental factors, coupled with the unique properties of cartilage tissue, underscores the need for continued research and innovation in this area. While advancements in tissue engineering and regenerative medicine offer hope for improved integration outcomes, effective solutions to this longstanding problem remain elusive.

#### Clinical Impact

Both surgical attachment and biological integration should be considered in a successful cartilage repair approach. In surgical attachment, the immediate physical securing of the graft or implant is often achieved using suturing techniques or applying adhesive materials. This process provides graft stability during the initial healing period. However, biological lateral integration is a gradual bonding process with adjacent native cartilage, which is mediated by molecular and cellular interactions and is essential for restoring functionality of the joint and long-term outcomes. Secure initial attachment supports effective biological integration.

## 5. Advantages and Disadvantages of Adhesive Hydrogel Scaffolds for Cartilage Repair

Adhesive hydrogel scaffolds offer several potential advantages for cartilage repair compared to traditional treatments and other biomaterial-based approaches:

### 5.1. Enhanced Tissue Integration

As mentioned hereabove, traditional treatments often fall short in addressing this critical aspect, leading to suboptimal outcomes [103,116,118]. For example, in microfracture surgery, the resulting repair tissue typically lacks the structural integrity and biomechanical properties of native cartilage due to inadequate integration with surrounding tissues. Similarly, in osteochondral allograft transplantation, integration between donor tissue and host cartilage may be compromised, leading to graft failure or delamination over time. Adhesive hydrogel scaffolds could potentially offer a solution to address this unmet need for enhanced tissue integration. By providing a stable and mechanically supportive environment, hydrogels could also minimize the formation of fibrous tissue. They may ensure secure anchoring of the scaffold within the defect site, minimizing the risk of delamination or displacement and facilitating the formation of durable cartilage tissue.

### 5.2. Improved Cell Retention and Viability

The adhesive properties of hydrogel scaffolds ensure efficient cell encapsulation and retention within the defect site, facilitating prolonged exposure to the local microenvironment. This enhanced cell retention promotes cell viability and functionality, maximizing the therapeutic potential of the implanted cells and facilitating tissue healing [119]. In treatments such as second-generation ACI, therapeutic cells are often injected into the cartilage defect using a liquid medium. However, this method can lead to significant cell leakage from the treated site, limiting the retention and viability of the implanted cells and reducing the efficacy of the treatment. Adhesive hydrogel scaffolds, on the other hand, provide a viscous medium that can confine cells within the defect zone, reducing the risk of cell leakage and enhancing their retention at the targeted site.

### 5.3. Tunable Properties

Hydrogel scaffolds offer tunable physical and biochemical properties [44]. They allow customization of scaffolds to match the mechanical and biological requirements of different cartilage defects. For example, in osteochondral allograft transplantation, the treatment relies on donor tissue to replace damaged cartilage, but the availability of suitable donor tissue is limited. Moreover, matching properties of donor tissue to recipient tissue can be challenging, leading to variability in outcomes and an increased risk of immune rejection or disease transmission.

### 5.4. Minimally Invasive Delivery

Traditional surgical procedures often require large incisions and extensive tissue dissection, leading to significant postoperative pain and prolonged recovery times. For instance, ACI involves multiple steps, including harvesting chondrocytes, culturing them ex vivo, and then re-implanting them into the defect site, requiring invasive steps such as membrane suturing and two separate surgeries [6]. In contrast, adhesive hydrogel scaffolds can be delivered via minimally invasive techniques, such as arthroscopy, which involve smaller incisions and reduced tissue trauma. Specifically, injectable and in situ curable hydrogels can be delivered directly into the cartilage defect through a small arthroscopic portal [64]. 

### 5.5. Biological Signaling

Hydrogel scaffolds can be engineered to deliver bioactive molecules, growth factors/cell sources, and signaling cues that promote tissue remodeling, angiogenesis, and anti-inflammatory responses. By modulating the local microenvironment, hydrogels facilitate the recruitment and differentiation of cells, further enhancing tissue repair and regeneration beyond what is achievable with conventional treatments.

### 5.6. Disadvantages of Hydrogel Scaffolds

Despite the unique advantages of hydrogels in cartilage repair, they may also have certain limitations that need to be considered. The encapsulation of cells in hydrogel networks may limit cell movement and proliferation and subsequently hinder the natural repair and integration with host tissue; therefore, a proper network design is essential. The degradation rate of hydrogels must be well controlled. Rapid degradation can result in losing their supportive function, while slow degradation may limit cell growth and regeneration. Variations in hydrogel preparation may lead to inconsistent and unreliable clinical outcomes. Also, the long-term effects and biocompatibility of some synthetic hydrogels are not fully understood, and therefore their safety and effectiveness should be further evaluated.

## 6. Adhesiveness Functionality and Quality Controls

Focusing on the adhesiveness feature and functionality of hydrogel systems, we review the considerations in production processes and how the adhesiveness attributes of hydrogel systems may be assessed. It includes in vitro testing methods, manufacturing in-process controls, and their potential to predict adhesiveness in clinical settings. We also emphasize the importance of demonstrating adhesiveness independently of biological payloads, which sets forth important product design and regulatory considerations.

### 6.1. Adhesiveness Assessment

Robust experimental setups for assessing adhesiveness are crucial to ensuring the reproducibility and high quality of adhesive hydrogel products. These assessment criteria encompass a comprehensive range of testing methods and in-process control measures, ensuring the reliability and performance of the final product.

#### 6.1.1. Mechanical Testing

**Figure 5:**
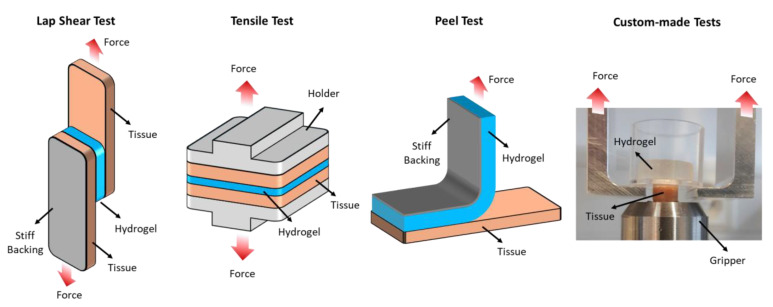
Mechanical adhesion testing methods, including lap shear, tensile, peel, and an example of a custom-made testing setup.

Adhesion relies on chemical, surface, and mechanical factors but is often assessed through mechanical tests. Standardized test methods provide a systematic approach to quantifying adhesive performance, enabling researchers to assess the effectiveness of different formulations and optimize product design. Various mechanical testing methods, including tensile [120,121], lap shear [44,122], peel [123,124], and custom-made testing [45,125], are employed to evaluate the hydrogel adhesive strength and durability (Figure 5).

Lap shear testing is a widely used method for evaluating the shear strength of adhesive bonds. In this test, two substrates bonded with the hydrogel adhesive are subjected to a shear force applied parallel to the adhesive interface. The force required to shear apart the bonded substrates provides a quantitative measure of the adhesive strength (i.e., the maximum force per unit area). Standard test protocols, such as ASTM F2255-05, provide guidelines for conducting lap shear tests and interpreting the results [126]. Peel testing evaluates the adhesive bond’s resistance to separation under tensile stress (i.e., the energy required to advance separation per unit area). Standard protocols such as ASTM D6862 (Standard Test Method for 90 Degree Peel Resistance of Adhesives) are commonly used peel test methods. Tensile testing evaluates the adhesive bond’s resistance to tensile forces, providing information on strength and elasticity. ASTM D897-08 (Standard Test Method for Tensile Properties of Adhesive Bonds) is an example of standard tensile test method applicable to hydrogel adhesives. While adherence to standard protocols ensures result reproducibility and comparability, customized test methods may be necessary for specific applications. These customized tests simulate real-life loading conditions, offering insights into adhesive performance. However, validation and correlation with established standards are essential for ensuring accuracy and reliability [45].

It should be noted that the biological complexity of tissue interfaces and the dynamic nature of physiological conditions may influence adhesive performance differently than in controlled laboratory settings. Therefore, validating in vitro findings with in vivo animal models is essential. Furthermore, the presence of biological payloads, such as cells or growth factors, can potentially enhance or alter the adhesive properties of a given hydrogel. Evaluating the adhesiveness of these materials without biological components can provide insights into their intrinsic properties, allowing assessment of whether the adhesive function remains effective even without additional bioactive elements.

#### 6.1.2. Physicochemical Characterization

Rheological analysis offers information on viscoelastic properties of hydrogel adhesives (e.g., viscosity, elasticity, etc.). For instance, optimized injectable hydrogel systems often exhibit a viscosity range between 1000 and 10,000 Pa and storage modulus (G’) values ranging from 10 to 100 kPa, depending on the formulation. By analyzing the rheological behavior of the hydrogel, especially for injectable hydrogel systems, it is possible to optimize the final formulation and processing parameters.

Bulk mechanical characterization is essential to determining the suitability of hydrogels for biomedical applications. Tensile testing evaluates the strength, elasticity, and deformation behavior of hydrogels by subjecting them to uniaxial tensile stress. This test measures parameters such as tensile strength, Young’s modulus, and elongation at break. Compression testing is also used to assess the behavior of hydrogels under compressive forces, critical for applications in load-bearing tissues such as cartilage. The stress-strain response from this test helps determine the hydrogel’s ability to withstand compressive loads.

### 6.2. In-Process Control Measures

In addition to endpoint mechanical testing, other in-process control measures, including real-time monitoring of critical process parameters and intermediate product testing, can be implemented to ensure reproducibility, quality, and safety of adhesive hydrogel/polymer production. Throughout the manufacturing process, this proactive approach aims to detect any deviations or anomalies that may impact the final product. Therefore, stringent controls over manufacturing parameters such as material composition, crosslinking density, and processing conditions are essential and allow manufacturers to identify and address potential issues before they impact product quality. Establishing current good manufacturing practices (cGMP) and implementing robust design controls help to minimize variability and ensure product uniformity.

#### 6.2.1. Chemical Characterization

Iterative monitoring of chemical composition and purity is essential to verifying the integrity of raw materials, intermediates, and final products. Techniques such as spectroscopy (e.g., FTIR, NMR), chromatography (e.g., HPLC, GC), and mass spectrometry can be employed to analyze the composition, molecular structure, and presence of impurities in the adhesive hydrogel/polymer. Adherence to GMP regulations ensures that chemical characterization processes comply with the applicable quality standards and regulatory requirements.

#### 6.2.2. Crosslinking Efficiency

Monitoring the crosslinking process is crucial for hydrogel-based adhesives to ensure proper gelation and structural integrity. Techniques such as gel permeation chromatography (GPC) or dynamic mechanical analysis (DMA) can be used to evaluate the degree of crosslinking, polymer network formation, and mechanical properties of the adhesive hydrogel during production.

#### 6.2.3. Sterility and Bioburden Control

Ensuring the sterility of adhesive hydrogels/polymer products is vital. Sterility testing, microbial enumeration, and endotoxin assays are conducted to verify compliance with microbial control standards and regulatory requirements for medical devices and implants. Regulatory bodies such as the FDA (U.S. Food and Drug Administration) and the European Medicines Agency (EMA), which are harmonized world-wide, provide sterility testing and microbial control guidelines in medical device manufacturing. Validated sterilization methods (e.g., gamma irradiation, ethylene oxide gas sterilization) and aseptic processing techniques can be implemented to ensure sterility. However, the material performance might be significantly affected after sterilization [127].

#### 6.2.4. Process Monitoring and Automation

Implementing real-time process monitoring and control systems allows for continuous surveillance of critical process parameters and adjustment of production variables. Automated feedback systems help to optimize process conditions and minimize batch-to-batch variability. ISO 13485 specifies requirements for quality management systems in the medical device industry, including process monitoring and control. ASTM E2500 provides guidelines for implementing risk-based approaches to process validation and automation in pharmaceutical manufacturing.

## 7. Clinical Translation and Regulatory Considerations

### 7.1. Biocompatibility and Safety Assessment of Adhesive Hydrogels

Adhesive hydrogel translation presents biocompatibility and safety assessment considerations due to their direct interaction with native cartilage tissue and surrounding joint environment. Since adhesive hydrogels are designed to interact closely with native tissue, compatibility with chondrocytes or other resident cells in cartilage should be evaluated to ensure minimal cytotoxicity and preservation of cellular functionality. Biocompatibility testing, including cytotoxicity, immunogenicity, and biodegradability assessments, is therefore essential. In vivo studies should assess the tissue response to adhesive hydrogels following implantation into cartilage defects. Histological analysis investigates the inflammatory responses, tissue integration, and foreign body reaction at the interface between the hydrogel scaffold and native tissue. Immunohistochemical staining for specific markers of cartilage regeneration, such as collagen type II and aggrecan, can further elucidate the biological response to the implanted scaffold. Since adhesive hydrogels are intended to degrade over time and be replaced by regenerated tissue, the biodegradability and biostability of the scaffold and its degradation products should be thoroughly evaluated. Degradation kinetics should be assessed to ensure controlled degradation and minimize adverse effects on surrounding tissues.

### 7.2. Preclinical Efficacy for Adhesive Hydrogels

Preclinical efficacy studies for adhesive hydrogels should focus on assessing their ability to promote tissue regeneration, facilitate tissue integration, and restore biomechanical function in cartilage defects. Animal models that closely mimic the anatomical and biomechanical properties of human joints, such as large animal models (e.g., sheep, goats), are preferred for preclinical studies to evaluate the performance of adhesive hydrogel scaffolds in cartilage repair [127,128,129]. Outcome measures should include macroscopic and histological evaluations of tissue regeneration, biomechanical testing of repaired tissue strength and integrity, and functional assessments of joint mobility and load-bearing capacity. Special attention should be given to evaluating the quality and durability of tissue integration between the hydrogel scaffold and native cartilage tissue.

### 7.3. Clinical Trial Design for Adhesive Hydrogels

Clinical trials in cartilage repair should be designed to evaluate the safety, efficacy, and clinical utility in human subjects presenting cartilage lesions. Patient selection criteria should consider factors such as lesion size, location, severity, and patient demographics to ensure the relevance and generalizability of trial results. The inclusion of patients with diverse cartilage defects and concomitant treatments can provide insights into the broader applicability of the scaffold. Outcome measures should include patient-reported outcomes (e.g., pain scores, functional assessments) and imaging modalities (e.g., MRI). Long-term follow-up assessments are critical for monitoring treatment durability, recurrence of symptoms, and adverse event occurrences over time.

### 7.4. Regulatory Approval Pathway for Adhesive Hydrogels

This procedure follows a phased approach, starting with preclinical testing and progressing through clinical trials to market authorization. Preclinical data should include comprehensive assessments of biocompatibility, safety, and efficacy specific to the adhesive hydrogel scaffold. Special considerations should be given to the degradation profile, tissue integration properties, and long-term effects of the scaffold on cartilage repair. Clinical trial protocols and study designs should be tailored to address the unique characteristics and performance attributes of the adhesive hydrogel scaffold. Regulatory submissions should provide detailed information on the scaffold’s composition, manufacturing process, performance characteristics, intended use, and clinical data supporting its safety and efficacy. Following regulatory approval, post-market surveillance and monitoring are essential for evaluating the long-term safety and performance of adhesive hydrogel scaffolds. Manufacturers should implement quality systems and adverse event reporting mechanisms to ensure ongoing compliance with regulatory requirements and standards [130].

Adherence to international standards is essential for regulatory compliance in developing and manufacturing hydrogel adhesive products. Compliance with these standards ensures that the product meets stringent safety and efficacy requirements, instilling confidence in their performance and reliability. Implementing design controls, as outlined in regulatory guidelines such as the FDA’s Quality System Regulation (21 CFR Part 820), is critical for establishing and maintaining the quality and consistency of hydrogel adhesive products. Design controls encompass the systematic identification of product specifications, risk management, and verification and validation testing, ensuring that the product meets its intended purpose and user requirements [131].

A comprehensive overview of the required considerations for the clinical translation of hydrogel products is presented in Table 6. It is important to note that adhesive hydrogels used in cartilage repair have additional specific considerations. Although the specific concerns for adhesive hydrogels may not be explicitly detailed as separate regulatory requirements, they are still integral to the overall regulatory evaluation. The unique properties of adhesive hydrogels, such as adhesion strength and mechanical properties, need to be thoroughly characterized and documented as part of the regulatory submission to demonstrate that the product is safe and effective for its intended use. These aspects of adhesive hydrogels are assessed indirectly through the required preclinical studies and bench tests mandated for regulatory submissions such as the FDA’s 510(k) or PMA, or the CE marking process in Europe. Furthermore, beyond meeting general biocompatibility standards, adhesive hydrogels must also address concerns related to the interaction between the adhesive properties and the tissue. This includes ensuring that the adhesive components do not cause irritation or adverse reactions in the surrounding tissue.

## 8. Future Perspectives and Conclusions

Integration of emerging technologies and clinical insights presents exciting opportunities for advancing the field of cartilage tissue engineering. Recent clinical observations highlight the need for improved therapeutic approaches for cartilage defects, particularly larger defects, where standard microfracture procedures may not suffice. Cell therapies such as autologous chondrocyte implantation (ACI) emerge as viable alternatives in such cases. However, the success of these therapies depends on the quality of the cell transplantation process, including prolonged retention and eventual engraftment at the defect site.

Hydrogel scaffolds, with their tunable properties and versatile design capabilities, can play a central role in the development of next-generation therapies for cartilage repair and regeneration. They could potentially offer durable and long-lasting solutions for patients with cartilage lesions.

Considering these clinical insights, there is a pressing need for formulation-based and protocol-oriented optimization of cell therapies similar to ACI. Current protocols in second-generation ACI involved the local injection of therapeutic chondrocytes suspended in a liquid medium, which can lead to significant cell leakage from the treated site. Moreover, it often has required additional steps such as membrane implantation to enhance cell retention.

To address these challenges, the localized injection of cells within an appropriate bioadhesive hydrogel matrix holds promise for enhancing therapeutic outcomes while simplifying the transplantation process. Hydrogels offer the advantage of providing a viscous medium that can confine cells within the defect zone, reducing the risk of cell leakage and enhancing their retention at the targeted site. Moreover, hydrogels can potentially exert ancillary therapeutic effects, such as promoting extracellular matrix synthesis, organization, and modulating paracrine signaling pathways, which are crucial for tissue regeneration.

However, specific functional properties are required for hydrogel-based approaches to realize their full potential in enhancing the efficiency of operative procedures and therapeutic outcomes. Cartilage adhesiveness is key to keeping the embedded cells securely at the targeted site and facilitating their integration with the surrounding tissue.

In conclusion, clinicians can offer advanced therapies by combining advances in cell sourcing, tissue engineering, and regenerative medicine, maximizing the chances of successful cartilage repair. Adhesive hydrogels offer a promising platform for addressing unmet needs in cartilage tissue engineering, particularly in promoting lateral integration with surrounding tissue. This can be achieved through careful design and optimization of hydrogel scaffolds, coupled with innovative strategies for enhancing cell-material interactions and tissue remodeling. By fostering interdisciplinary collaborations and embracing emerging technologies, we can realize the full potential of hydrogel-based therapies for improving patient outcomes and quality of life.

## Figures and Tables

**Table 1:** Historical comparison of existing treatments for cartilage repair, including their respective advantages, limitations, and implications for tissue integration.

Treatment Method	Advantages	Limitations	Integration Mechanisms	Implications and Factors Contributing to Weak Integration in Early Phases	References
Bone Marrow Stimulation	- Minimally invasive procedure - Cost-effective - Suitable for small defects	- Fibrocartilage rather than hyaline cartilage - Limited durability and long-term efficacy - Not suitable for large defects	- Clot formation and recruitment of mesenchymal stem cells	- Immature tissue formation and limited matrix deposition - Fibrocartilage formation may compromise mechanical properties and long-term function	[3,23,24]
Osteochondral Autograft Transfer (OATS) and Mosaicplasty	- Utilizes patient’s own osteochondral tissue - Structural support and immediate stability	- Limited availability of donor tissue - Risk of donor site morbidity - Not suitable for larger defects	- Integration through precise graft matching - Promotion of chondrocyte migration and matrix production - Bone-to-bone fusion	- Challenges in achieving seamless integration between graft and host tissue - Insufficient graft-host tissue congruency - Inadequate cell migration and matrix production	[25,26,27]
Osteochondral Allograft Transplantation	- Provides mature, hyaline-like cartilage - Suitable for larger defects - Eliminates risk of donor site morbidity compared to autografts	- Limited availability of matching grafts - High cost - Requires matching of graft size and contour - Requirement to implant the graft within 28 days	- Integration through precise graft matching - Promotion of chondrocyte migration and matrix production - Bone-to-bone fusion	- Requires adequate host tissue preparation for successful integration - Insufficient graft-host tissue matching - Inadequate cell migration and matrix production	[28,29,30]
First- and second-generation ACI	- Potential for hyaline-like cartilage formation	- Limited availability of healthy chondrocytes for implantation - Risk of cell leakage - Further tissue damage by suturing the membrane	- Chondrocyte proliferation and matrix production - Gradual infiltration of native cells and matrix from surrounding tissue	- Limited cell retention and survival in the defect area - Inadequate cell migration and matrix production - Challenges in achieving uniform integration with surrounding tissue	[25,26]
Third-generation ACI	- Improved cell retention and distribution within cell carriers - Early cell differentiation using pre-seeded scaffolds	- Limited availability of healthy chondrocytes for implantation - Higher cost compared to traditional ACI	- Chondrocyte proliferation and matrix production within the scaffold - Gradual infiltration of native cells and matrix from surrounding tissues	- Inadequate cell migration and matrix production - Scaffold degradation may affect tissue integration - Suboptimal extracellular matrix production	[25,26,31]

**Table 4:** Mechanisms impeding lateral integration in cartilage repair.

Mechanism	Contributing Parameters/Attributes	Examples/Previous Evidence
Cellular factors	Chondrocyte viability: Cell death hinders integration between neo-cartilage and existing tissue.	- Significant cell death reported at the interface between host and repaired tissue in partial-thickness chondral defects. - In vitro wounding induces a zone of cell death characterized by necrosis and apoptosis.
	Chondrocyte phenotype: Dedifferentiation during expansion compromises chondrocyte function.	- Dedifferentiated chondrocytes show limited redifferentiation capacity, affecting integration. - Incomplete redifferentiation can compromise normal chondrocyte function.
Donor-related factors	Donor age: Age-related decline in chondrocyte function impedes integration.	- Age-related reductions in chondrocyte function affect repair outcomes. - Young tissue exhibits better repair and integration outcomes compared to aged tissue.
	Developmental origins: Differences in tissue origin affect biosynthetic capacities and matrix production.	- Tissues from different developmental origins may have varied integration capacities. - Mixing tissues from different origins may or may not result in segregation.
Extracellular matrix factors	Collagen network: Collagen deposition and crosslinking influence integration.	- Collagenase treatment enhances integration by promoting collagen deposition and chondrocyte migration. - Lysyl-oxidase-mediated crosslinking affects fusion between cartilages.
	Proteoglycans: The presence of proteoglycans inhibits chondrocyte migration and integration.	- Enzymatic removal of proteoglycans increases chondrocyte mobility and enhances integration. - Loss of proteoglycans using chemical crosslinkers enhances adhesion of cartilage surfaces.
Biomaterials and scaffold integration	Low adhesion performance: Inadequate scaffold adhesion affects tissue integration.	- Scaffolds with poor adhesion may fail to properly integrate with surrounding cartilage. - Low adhesion can result in delamination of the repaired tissue from the host cartilage.
	Inappropriate mechanical properties: Scaffold properties may not match physiological requirements, impacting integration.	- Scaffolds with mismatched mechanical properties may lead to mechanical failure and hinder integration. - Biomechanically weak scaffolds may collapse under load, preventing integration.
	Inadequate biocompatibility: Scaffold materials may elicit immune responses or cytotoxic effects, impeding integration.	- Biocompatibility issues with scaffold materials can lead to inflammation and hinder tissue integration. - Cytotoxicity of scaffold components may impair chondrocyte function and integration.
	Insufficient porosity: Low porosity limits cell infiltration and nutrient exchange, affecting integration.	- Scaffolds with inadequate porosity may restrict cell migration and proliferation, hindering tissue integration. - Poor nutrient exchange due to low porosity can impair cell viability and integration.

**Table 5:** Strategies for enhancing lateral integration in cartilage repair.

Parameter	Potential Strategy	Examples/Previous Evidence
Cellular factors	Promote chondrocyte viability: Use of caspase inhibitors to inhibit apoptotic cell death	Inhibition of apoptotic cell death using caspase inhibitors such as ZVAD-fmk has shown partial rescue of cell death and enhancing lateral integration [110].
Utilization of young tissues	Utilizing tissues from younger donors: Higher biosynthetic capacities and integration potential	Transplantation of embryonic tissues into defects in mature animals has shown improved restoration of surface continuity and lateral integration [111].
External stimuli and treatments	Use of growth factors: Controlled release to promote chondrogenesis and tissue integration	Use of platelet-rich plasma (PRP) as a growth factor blend, induced better graft integration [112].
	Mechanical stimulation	Spinner bioreactor stimulation enhanced integration, boosting collagen content and gene expression related to integration. Early loading post-surgery could improve cartilage integration [113].
Extracellular matrix factors	Modulate collagen network: Use of collagen crosslinking inhibitors to enhance fusion.	Inhibition of lysyl-oxidase-mediated collagen crosslinking accelerated collagen maturation and increased adhesive strength, promoting integration [114].
	Manipulate proteoglycan content: Enzymatic removal of proteoglycans to promote chondrocyte mobility.	Enzymatic removal of proteoglycans increased chondrocyte mobility and enhanced integration [115].
Biomaterials and scaffold integration	Scaffold adhesion	An intrinsically adhesive hydrogel demonstrated tissue integration after two days of in vivo implantation in cartilage defects [44].
	Optimal porosity	Allowing better cell infiltration and nutrient exchange, enhancing integration [116].
Surface modification	Bioadhesive glues and bridging polymers (e.g., fibrin, etc.)	Employing chondroitin sulfate (CS) functionalized with methacrylate and aldehyde groups facilitated mechanical stability for tissue repair [117].

**Table 6:** Overview of key evaluation parameters, methods, and regulatory considerations for cell-based and non-cell-based adhesive hydrogel scaffolds in cartilage repair.

Consideration	Device Category	Description	Evaluation Method	Standards/References
**Biocompatibility Assessment**	Cell-Based	- Assessment of cell viability, proliferation, and differentiation within the hydrogel scaffold in vitro. - Evaluation of host immune response and tissue integration post-implantation.	- Live/dead staining, MTT assay, Alamar Blue assay for cell viability. - Immunohistochemistry for cell-specific markers (e.g., collagen type II, aggrecan) for differentiation. - ELISA for evaluation of inflammatory cytokines (e.g., TNF-α, IL-6) post-implantation.	- ISO 10993 series for biocompatibility testing. - ASTM F1903-98 for evaluation of tissue-engineered cartilage constructs.
	Non-Cell-Based	- Examination of tissue response and integration without cellular components. - Focus on minimizing inflammatory reactions and promoting tissue regeneration.	- Histological analysis (e.g., H and E staining) for tissue response and integration. - Immunohistochemistry for ECM components (e.g., collagen type II, glycosaminoglycans).	- ISO 10993 series for biocompatibility testing. - ASTM F2150-18 for standard guide for tissue-engineered medical products (TEMPs).
**Preclinical Efficacy Studies**	Cell-Based	- Demonstration of chondrogenic potential and matrix synthesis by seeded cells. - Evaluation of scaffold degradation and tissue remodeling.	- Immunohistochemistry for chondrogenic markers (e.g., collagen type II, aggrecan). - Biochemical assays (e.g., GAG/DNA content, hydroxyproline assay) for matrix synthesis. - SEM and mechanical testing for scaffold degradation and mechanical properties.	- ASTM F2451-05 for testing the mechanical properties of hydrogels for cartilage repair. - ISO 10993 series for biocompatibility testing.
	Non-Cell-Based	- Emphasis on scaffold stability, mechanical properties, and biodegradation characteristics. - Assessment of tissue ingrowth and integration with surrounding cartilage.	- Mechanical testing (e.g., tensile, compressive, shear) for scaffold stability and properties. - Histomorphometry for tissue ingrowth and integration.	- ASTM F2451-05 for testing the mechanical properties of hydrogels for cartilage repair. - ISO 10993 series for biocompatibility testing.
**Clinical Trial Design**	Cell-Based	- Consideration of cell sourcing, expansion, and delivery methods. -Evaluation of cell retention, survival, and functionality post-implantation.	- In vivo imaging techniques (e.g., MRI, CT) for cell tracking and localization. - Biopsies for histological evaluation of cell survival and phenotype. - Functional assessments (e.g., joint function scores, pain scales) for therapeutic outcomes.	- FDA Guidance for Industry: Preclinical Assessment of Investigational Cellular and Gene Therapy Products (FDA-2012-D-1038). - EMA Guideline on Human Cell-Based Medicinal Products [132].
	Non-Cell-Based	- Simplified trial design without the complexity of cell handling and processing. - Focus on scaffold delivery, integration, and therapeutic outcomes.	- In vivo imaging techniques (e.g., MRI, CT) for scaffold localization and integration. - Functional assessments (e.g., joint function scores, pain scales) for therapeutic outcomes.	- FDA Guidance for Industry: Considerations for the Design of Early-Phase Clinical Trials of Cellular and Gene Therapy Products. - Regulation (EU) 2017/745: New Medical Device Regulation (MDR).
**Regulatory Approval Pathway**	Cell-Based	- Additional regulatory scrutiny for cell sourcing, processing, and manipulation. - Compliance with Good Manufacturing Practice (GMP) standards for cell-based therapies.	- Adherence to GMP regulations for cell isolation, expansion, and manipulation. - Documentation of cell identity, purity, and potency. - Validation of manufacturing processes and quality control measures.	- FDA Guidance for Industry: CGMP for Phase 1 Investigational Drug and Biological Products. - EMA Guidelines on Good Manufacturing Practice Specific to Advanced Therapy Medicinal Products [133].
	Non-Cell-Based	- Streamlined regulatory pathway focusing on scaffold composition, manufacturing, and performance. - Emphasis on biocompatibility, safety, and efficacy of the scaffold material.	- Compliance with regulatory guidelines for medical devices (e.g., ISO 13485). - Documentation of material characterization, sterilization, and biocompatibility testing.	- FDA Guidance for Premarket Approval (PMA) or Premarket Notification 510(k) depending on device classification. - ISO 13485:2016 for quality management systems for medical devices.

## Data Availability

The data and information related to this work are freely available within the article files.
